# Solar Cells for Indoor Applications: Progress and Development

**DOI:** 10.3390/polym12061338

**Published:** 2020-06-12

**Authors:** Swarup Biswas, Hyeok Kim

**Affiliations:** School of Electrical and Computer Engineering, Institute of Information Technology, University of Seoul, 163 Seoulsiripdaero, Dongdaemun-gu, Seoul 02504, Korea; biswas1988@uos.ac.kr

**Keywords:** internet of things, microelectronic device, low-intensity indoor light, photovoltaic technology

## Abstract

The Internet of things (IoT) has been rapidly growing in the past few years. IoT connects numerous devices, such as wireless sensors, actuators, and wearable devices, to optimize and monitor daily activities. Most of these devices require power in the microwatt range and operate indoors. To this end, a self-sustainable power source, such as a photovoltaic (PV) cell, which can harvest low-intensity indoor light, is appropriate. Recently, the development of highly efficient PV cells for indoor applications has attracted tremendous attention. Therefore, different types of PV materials, such as inorganic, dye-sensitized, organic, and perovskite materials, have been employed for harvesting low-intensity indoor light energy. Although considerable efforts have been made by researchers to develop low-cost, stable, and efficient PV cells for indoor applications, Extensive investigation is necessary to resolve some critical issues concerning PV cells, such as environmental stability, lifetime, large-area fabrication, mechanical flexibility, and production cost. To address these issues, a systematic review of these aspects will be highly useful to the research community. This study discusses the current status of the development of indoor PV cells based on previous reports. First, we have provided relevant background information. Then, we have described the different indoor light sources, and subsequently critically reviewed previous reports regarding indoor solar cells based on different active materials such as inorganic, dye-sensitized, organic, and perovskite. Finally, we have placed an attempt to provide insight into factors needed to further improve the feasibility of PV technology for indoor applications.

## 1. Introduction

Currently, the demand for self-powered microelectronic indoor devices, such as sensors, smart meters, wearable devices, and actuators, is steadily increasing, as they make daily activities faster by automating them through the Internet of things (IoT) technology [1,2,3,4,5]. It is expected that billions of interconnected wireless sensors, personal data storage devices, actuators, and household products will be installed by 2020 through IoT technology [6]. Wired power sources or normal batteries are not suitable for these low-power devices. Moreover, daily charging or frequent replacement of batteries is not practical in small wireless devices [7]. Therefore, alternative microscale ambient-energy-harvesting technologies, such as thermoelectric generators [8,9], mechanical energy harvesters [10,11], and low-intensity light energy harvesters [12,13], can be an excellent option for powering small wireless devices. Interestingly, most of the IoT devices are operated indoors. To this end, light energy is an attractive energy source, owing to its ambient availability and easy accessibility [14,15]. A photovoltaic (PV) cell converts indoor light energy into electrical energy. Consequently, a PV cell can be a suitable option for solving the hardware-related (powering) problem of future wireless sensor networks. There is a difference between the irradiances of light in outdoor and indoor environments. The source of light in an outdoor environment is the sun and its irradiance intensity is very high (100 mW/cm^2^), whereas the irradiance intensity of indoor light is 10–1000 times lower compared with that of sunlight [16]. In addition, indoor illuminating systems are more complex than outdoor systems. Moreover, the irradiance power intensity and luminance of indoor light can vary with time and the nature of the source [17,18]. During the daytime, a room can be illuminated by sunlight entering through the window or light from different artificial light sources (light-emitting diode (LED) lamps, sodium lamps, fluorescent tube lights, bulbs, etc.), or by a combination of these two sources; in contrast, only artificial sources can illuminate a room at nighttime.

In the past few years, the development of PV cells specifically designed for harvesting low-intensity diffused indoor light energy has attracted the interest of researchers [19,20,21,22,23]. Various PV materials have been employed so far to develop efficient solar cells for indoor applications. These solar cells can be classified into four different categories, namely, inorganic solar cells (ISCs) [14,24,25], dye-sensitized solar cells (DSSCs) [21,26,27,28,29,30,31], organic solar cells (OSCs) [13,16,32,33,34,35,36,37,38,39,40], and perovskite solar cells (PVSCs). Among them, ISCs exhibit the highest power conversion efficiency (PCE) in outdoor environments (1-sun condition), whereas DSSCs, OSCs, and PVSCs show a good performance in indoor environments (for low-intensity light). Interestingly, DSSCs for indoor applications are already available in the market, owing to their high PCE under the illumination of low-intensity light. It is evident that solar cells based on soft materials, such as OSCs, DSSCs, and PVSCs, have better applicability for indoor applications because of their mechanical flexibility, low weight, good spectral matching with indoor light sources, high open-circuit voltage, and high PCE. However, to date, very few studies have been conducted to standardize their different aspects for commercialization [41,42,43]. The performance of solar cells depends on various factors, such as device architecture, the nature of the active material, module spectral response, the power intensity and illuminance of the light source, irradiance, temperature, and reflection. Significant efforts have been made by researchers in the past few years to develop low-cost, stable, and efficient solar cells for indoor applications; however, such efforts are still insufficient [42]. Further investigation is required to resolve some critical issues in solar cells, such as environmental stability, lifetime, large-area fabrication, mechanical flexibility, and spectral matching between the absorbance spectra of the solar cell active material and the irradiance spectra of the indoor light source. In this regard, a detailed review of the development of solar cells for indoor applications can be highly useful to examine the different aspects that require proper attention. This study attempts to provide a detailed review of the development of indoor solar cell technology. First, we discuss the different indoor light sources. Subsequently, previous reports concerning indoor solar cells based on different active materials are critically reviewed. Through this review, we analyze the various aspects that should be considered for the development of indoor solar cell technology.

## 2. Sources of Indoor Light

**Figure 1:**
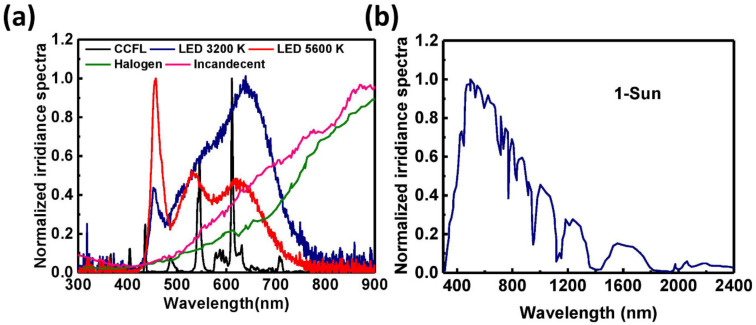
(**a**) Irradiance spectra of different indoor light sources. (Reproduced from [13,34], with permission from Elsevier, 2019 and Elsevier, 2016 respectively); (**b**) Solar spectra (AM1.5G). (Reproduced from [44], with permission from Elsevier, 2006).

**Figure 2:**
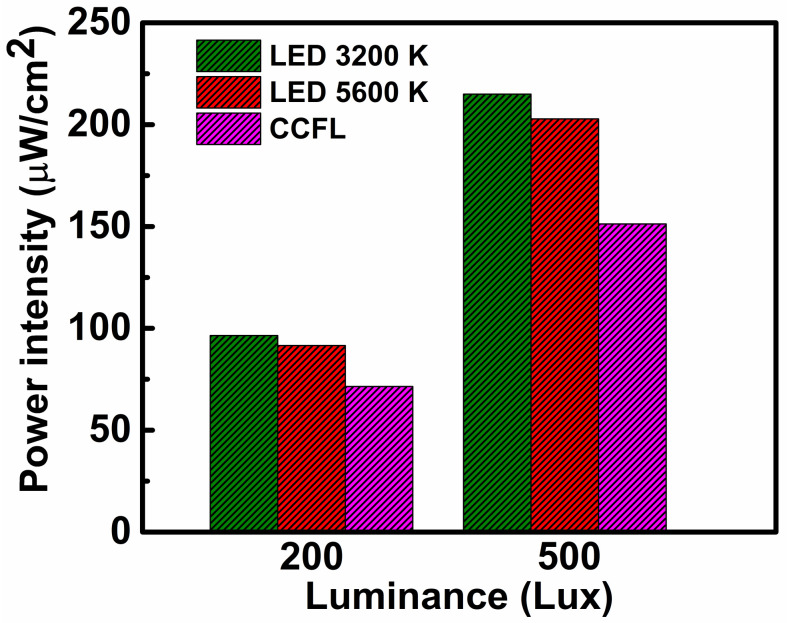
Comparison of irradiance power intensity of different light sources at a fixed luminance value.

A light source is a crucial part of an indoor light energy harvesting system [19]. Various types of light sources are used as illuminating agents in a room. Among them, LED bulbs, cold cathode fluorescent lamp (CCFL) bulbs, halogen bulbs, and incandescent bulbs are the most commonly used light sources for indoor environments [42]. Interestingly, the aforementioned artificial light sources have different types of irradiance spectra (Figure 1a). From Figure 1a, it can be observed that the irradiance spectrum of the fluorescent tube light has peaks at ~400 nm and ~600 nm, whereas that of the white LED light has peaks at ~450 nm and ~550 nm. In contrast, the irradiance spectra of halogen and incandescent bulbs exhibit a gradually increasing trend within the wavelength range of 300–900 nm. Also, the wavelength distribution of the light generated from the different sources is not the same. The LED light source has a wider and more continuous energy distribution than that of a fluorescent lamp. In addition, the light generated from the various light sources differs in terms of quality (these sources produce light with different irradiance power intensities at the same luminance value). Figure 2 presents the variation of irradiance power intensity (measured within the wavelength range of 300–900 nm) of the different light sources at a particular luminance value. In Figure 2, it can be observed that the irradiance power intensity of the LED 3200 K lamp is 96.4 µW/cm^2^ at a luminance of 200 lx, whereas the irradiance power intensities of the LED 5600 K bulb and CCFL bulb are 91.60 µW/cm^2^ and 71.49 µW/cm^2^, respectively, at the same luminance level.

Consequently, solar cells usually exhibit different spectral responses to different light sources at a fixed luminance value. This phenomenon can result in different amounts of light energy harvested by a solar panel operating under different artificial light sources at a fixed luminance value. Therefore, the fabrication of specially designed solar cells for indoor applications is not an easy task. Different parameters of solar cells must be optimized for indoor light conditions. 

The device should be designed in such a manner that it can operate efficiently under the illumination of the most commonly used indoor light sources. In the last few years, the optimization of various device parameters of solar cells for indoor applications and the development of synergic semiconducting materials (having wider absorption spectra that can be well-matched with the irradiance spectra of commonly used artificial light sources) as active layers of solar cells have become the main objectives of researchers in fabricating a highly efficient solar cell for indoor applications.

## 3. PV Cells for Indoor Applications

**Figure 3:**
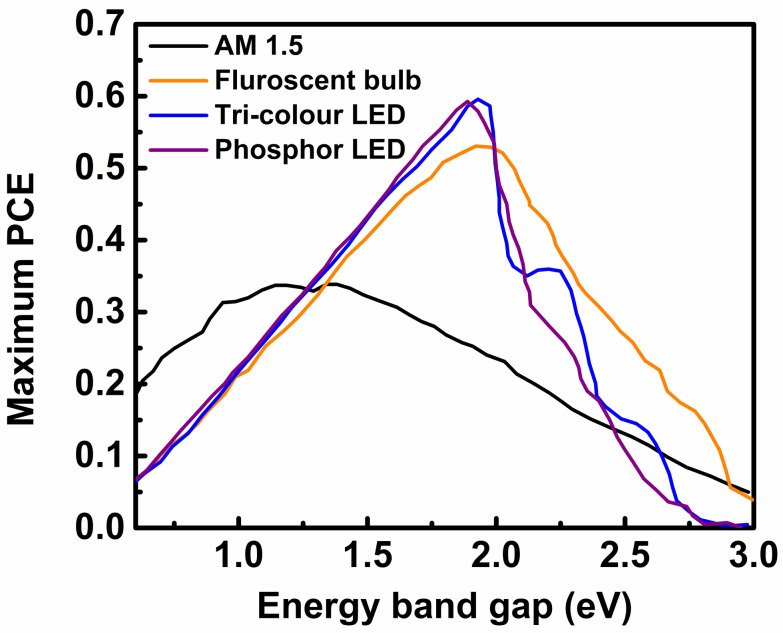
Variation of the estimated maximum PCE with the bandgap energy values of various materials for different illuminating agents. (Reproduced from [46], with permission from Wiley, 2013).

The PV materials used for normal solar cells have wide absorption spectra because the irradiance spectrum of sunlight is wide and expands from the ultraviolet (300 nm) to the near-infrared (2500 nm) region (Figure 1b). However, the irradiance spectra of commonly used indoor light sources have high-energy photons and are narrow (300 nm to 1000 nm) (Figure 1a). Therefore, to avoid unexpected energy losses due to thermalization and non-absorption of light, the PV materials used for indoor solar cells should have narrow absorption bands [15]. It has been determined from theoretical calculations that the optimal energy band gap of an ideal PV material for fabricating the most efficient indoor solar cell is approximately 1.9 eV [45,46]. In contrast, the optimized energy bandgap of PV materials for a normal solar cell is approximately 1.35 eV [15,47] (Figure 3). Considering this important factor, solar cells based on different types of PV materials have been studied over the last decade, typically under the illumination of indoor light for developing efficient, flexible, durable, and stable indoor PV devices. PV materials can be classified into four categories, namely, inorganic, dye-sensitized, organic, and perovskite materials. The recent development of indoor solar cells based on these PV materials is discussed below.

### 3.1. Solar Cells Based on Inorganic Materials for Indoor Applications

**Figure 4:**
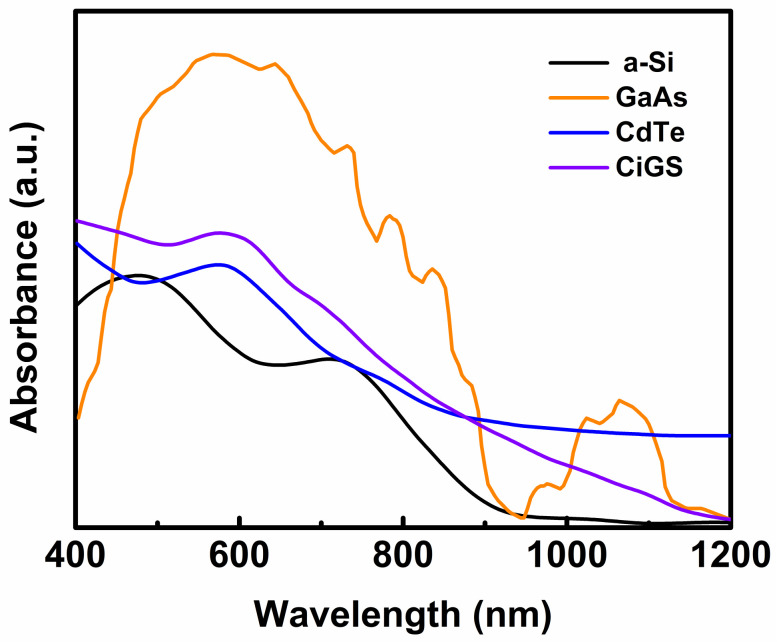
Absorption spectra of some widely used active materials in ISCs for indoor applications (Reproduced from [60,61], with permission from Elsevier, 2016 and AIP, 2016 respectively).

Solar cells based on inorganic semiconducting materials are called first-generation solar cells. The first research article on a silicon-based solar cell was published in 1941 [48]. Subsequently, various research groups have attempted to improve the applicability, durability, PCE, and lifetime of silicon-based solar cells through different techniques [49]. Through continuous efforts, the crystalline silicon (c-Si) solar cell has achieved a PCE of 26% when operating under the 1-sun condition [50,51]. In the early age of indoor solar cells (around 1970), amorphous silicon (a-Si) PV cells were used to harvest indoor light energy for powering various portable devices, such as calculators and watches [52]. However, the device efficiency was low and the production cost was high at that time. Therefore, researchers have focused their attention on the development of efficient, environmentally stable, and low-cost new PV materials for fabricating highly efficient new-generation solar cells for indoor applications. In addition to the development of new inorganic PV materials for indoor solar cells, some researchers have attempted to improve the performance and mechanical flexibility of Si-based indoor solar cells through some special techniques. In 2014, Foti et al. fabricated an inorganic PV material (hydrogenated a-Si)-based flexible solar cell on a polymeric substrate for indoor applications [53]. Their device exhibited highly satisfactory flexibility and stability. The device could harvest indoor light (F12) energy with a PCE of ~9%. Águas et al. also developed a thin-film Si-based solar cell on a paper substrate for indoor applications [54]. Furthermore, Kao and co-workers reported that the introduction of hydrogenated a-Si carbide in an a-Si thin-film solar cell could enhance the PCE of the device by up to 9.6% under the illumination of a 500 lx LED lamp [55]. From an extensive review, it has been observed that the Si-based solar cell can operate with high efficiency (PCE = 26%) under the 1-sun condition because of its broad absorption spectrum (Figure 4). However, it cannot efficiently harvest ambient light energy (currently, the maximum reported indoor PCE is approximately 9.6%) because of its low bandgap energy (~1.1 eV). In addition to the classical Si, researchers have employed other inorganic PV materials, such as III–IV semiconductors [14], CdS/CdTe [56], and CIGS [57,58,59], for developing indoor solar cells. The III–IV compound semiconducting materials are of significant interest; they have a high and tunable bandgap energy, which renders them suitable PV materials for indoor solar cells [15]. 

However, due to their high prices, the III–IV compound semiconducting materials have not been utilized for the large-scale production of solar panels and they are only affordable for fabricating small indoor PV modules. For the past few years, researchers have made significant efforts toward the development of various indoor solar cells based on III–IV semiconducting materials. Gallium arsenide (GaAs) is one of the most widely used III–IV semiconducting compound materials for PV applications, owing to its high charge carrier mobility and direct bandgap [25]. The PCE of a single-junction solar cell based on a GaAs PV material has already reached approximately 30% for the 1-sun condition [50]. The bandgap energy of a normal GaAs is ~1.42 eV; however, the ideal bandgap energy of PV materials for indoor solar cells is ~1.9 eV [14]. Interestingly, the bandgap energy of GaAs can be increased through doping [14]. In this regard, Teran and co-workers enhanced the bandgap energy of GaAs from 1.42 eV to 1.67 eV by controlled doping with Al [15]. Furthermore, a solar cell based on an Al_0.20_Ga_0.80_As PV material exhibited a PCE of ~21% under the illumination of a 580 lx LED lamp, whereas the undoped GaAs-based solar cell exhibited a PCE of only ~15% under the same illuminating condition [15]. In 2015, Mathews et al. reported that a credit-card-sized solar cell based on a GaAs or GaInP semiconducting compound material could harvest power of ~4 mW under 1000 lx LED and compact fluorescent lamps [14]. In addition to the III–IV semiconducting compound materials, other inorganic semiconducting materials such as CdS/CdTe and CIGS have been employed for indoor applications; however, the devices based on these materials have exhibited a poor PCE in indoor environments. For example, a CdS/CdTe-based solar cell exhibited a PCE ~22% under the 1-sun condition but the same solar cell exhibited a PCE only 8% under the illumination of low-intensity indoor light [56]. In contrast, a CIGS-based solar cell showed a PCE ~22% under the 1-sun condition; however, its performance was extremely poor (PCE ~2.64%) under the illumination of low-intensity LED light [62]. 

Some significant results on different ISCs for indoor applications are summarized in Table 1. From this systematic review on indoor solar cells based on inorganic materials, it is evident that among various inorganic PV materials, the III–IV semiconducting compound materials are the most preferable for indoor solar cells owing to their high efficiency, good spectral matching (Figure 4), and environmental stability. In this regard, a doped GaAs-based indoor solar cell has already achieved a PCE ~30% under the illumination of a low-intensity LED lamp in a laboratory. Furthermore, the bandgap energy and other properties of such materials, such as electrical conductivity, transparency, surface morphology, and absorption spectrum (Figure 4), can be fully modified via controlled doping. The only drawback is the production cost. Therefore, further studies on these types of inorganic semiconducting compounds are necessary for tuning their different physicochemical properties according to the requirements and reducing their production cost.

### 3.2. DSSCs for Indoor Applications

**Figure 5:**
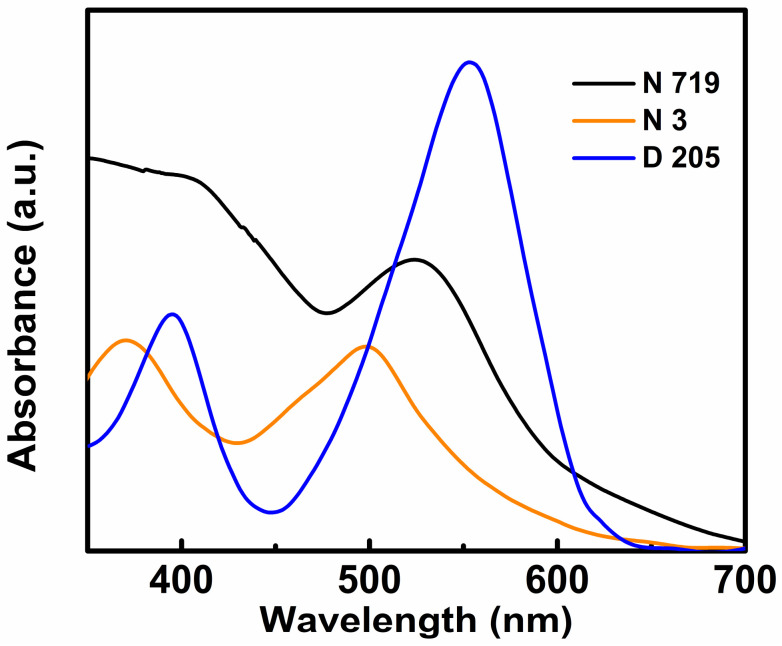
Absorption spectra of some widely used dyes in DSSCs for indoor application. (Reproduced from [69,70], with permission from RSC Publishing, 2011 and ACS Publications, 2009 respectively).

The first report on DSSCs was published by Grätzel and O’regan in 1991 [63]. Subsequently, various research groups have contributed to the improvement of different aspects, such as the design optimization, PCE, mechanical and environmental stability, and fabrication techniques of DSSCs [64]. After almost 30 years, a PCE of only 12.3% has been achieved (NREL best research cell efficiency table) from DSSCs at the 1-sun condition. This may be due to their shorter absorption band. However, a different result was obtained when the DSSCs were tested under low-intensity indoor light. The devices exhibited very high PCE values, owing to their unique absorption spectra (generally expanded within 300–800 nm) [65,66,67,68] (Figure 5). 

**Figure 6:**
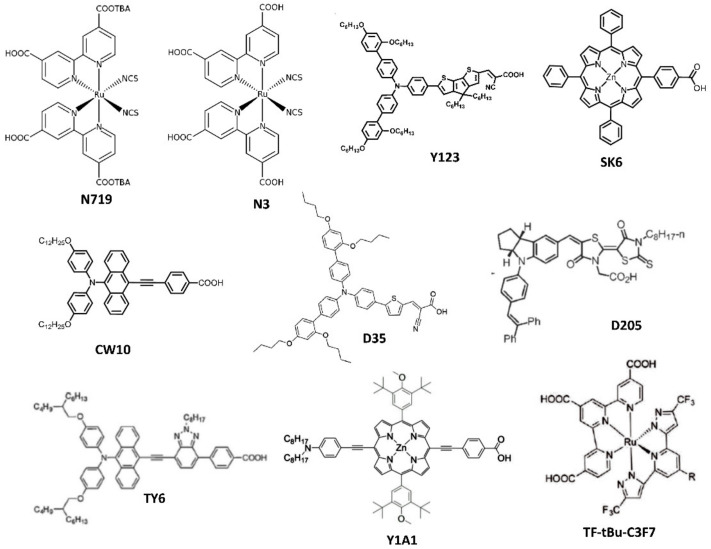
Chemical structures of various widely used dye sensitizers for indoor DSSCs.

The first laboratory trial on DSSCs has been reported in 2000 by Grätzel et al. The authors fabricated an N719 dye-based DSSC and tested it under the illumination of a Philips TLD 840 fluorescent lamp. The device harvested power of 69.8 μW/cm^2^ for a luminance of 250 lx. Furthermore, the device exhibited high stability for low-power applications [71]. Subsequently, various research groups have studied DSSCs for different dyes such as N719 [72], N3 [73], CW10 [74], Y1A1 [75], D205 [72], Y123 [72], TF-tBu-C3F7 [76], TY6 [31], SK6 [74], SK7 [77], D35:XY1 [21], XY1b:Y123 [78], L350 [79], MD5 [80], MD7 [80], MM-6/MM-3 [81], LI-130 [82], SK6+CW10 [74], and XY1 [83] for indoor applications. The chemical structures of some dyes are depicted in Figure 6. Among those dyes, N719, N3, and TF-tBu-C3F7 are ruthenium (Ru)-based dyes. The successful utilization of N719-dye-based DSSCs in indoor light energy harvesting systems has motivated researchers to develop new device fabrication technologies, device architectures, and Ru-based dyes for fabricating DSSCs with better performance for indoor applications. For example, Lan et al. improved the PCE of an N719 based DSSC for low-intensity light by optimizing the concentration of the electrolyte [84]. The authors observed that the concentration of iodine within the electrolyte of a DSSC had a significant impact on the performance of the device for indoor applications and a low amount of iodine could enhance the PCE of the DSSC through the reduction of electron recombination at the interface of titanium dioxide/dye/electrolyte. In 2013, a succinonitrile-based solid-state electrolyte was used by Byrne et al. in an N719 DSSC [85]. The device was tested under the illumination of a fluorescent bulb (200 lx), and it could harvest power of 1.63 µW from the ambient light energy. He et al. fabricated a platinum-free N719 based DSSC [86] using graphene dot/ poly (3,4-ethylenedioxythiophene) (PEDOT) polystyrene sulfonate (PSS) as a counter electrode. Their device exhibited moderate PCE under the illumination of low-intensity indoor light. Pt is an expensive material and it often reacts with electrolytes; therefore, for the past few years, some researchers have also attempted to develop a low-cost, less reactive, and stable alternative material for a DSSC counter electrode (specially designed for indoor applications) [87,88,89]. In 2016, Kapil et al. fabricated Ti-coil-based cylindrical DSSCs by using different dyes such as N719, D205, and Y123 [72]. The devices were tested under the illumination of both 1-sun and fluorescent light. The N719 dye-based DSSC exhibited better performance than the other two dye-based DSSCs under both illuminating conditions.

Wen et al. fabricated and utilized a polyvinylidene-fluoride-based quasi-solid electrolyte for an N719-dye-based DSSC. Their device exhibited better stability than a liquid-electrolyte-based DSSC for both indoor and outdoor conditions [90]. In 2017, Chang and Chi prepared a Ru-based new dye sensitizer TF-tBu-C3F7 [76]. Further, they utilized the dye in a DSSC, which exhibited PCE of 20.37% and 16.05% under the illumination of 2400 lx T5 fluorescent lamp and LED lamp, respectively. Wang and Teng could increase the PCE of an N3-dye-based solar cell to approximately 23% for indoor applications through optimal doping of its TiO_2_ layer by zinc [91]. They proposed that an optimized amount of the doping reagent (Zn) reduces the decay of the energy efficiency (photo to electrical) of the device under the illumination of low-intensity light. Ru is a rare transition metal and its extraction from nature is difficult. Therefore, for the past few years, some researchers have also attempted to develop Ru-free dyes such as Y1A1, SK6, SK7, D35:XY1, TY6, MD5, MD7, MM-3, MM-6, L350, CW10, LI-130, Y123, XY1b:Y123, D205, and Y123 for solar cell applications. Chen and co-workers used two types of Ru-free dyes, namely, SK6 (porphyrin-based) and CW10 (anthracene-based), in a DSSC for indoor applications [74]. The CW10-dye-based DSSC exhibited PCE of 22.5% and 20.90% under the illumination of T5 fluorescent and LED lamps, respectively. This team synthesized two other Ru-free dyes, i.e., SK7 and YD2, and utilized them in a DSSC. The SK7-based DSSC exhibited PCE of 19.72% and 15.54% under the illumination of T5 fluorescent and LED lamps, respectively. In contrast, the YD2-dye-based DSSC showed PCE of 20.00% and 16.57% under the illumination of the T5 fluorescent and LED lamps, respectively [92]. Liu et al. demonstrated the influence of a compact blocking layer on the performance of a Y123-dye-based solar cell for indoor applications [93]. They observed that the presence of a compact blocking layer can reduce the leakage of electrons over a wide range of intensities. This phenomenon can improve the PCE of a DSSC in an indoor environment. Venkatesan and co-workers fabricated a highly efficient (PCE values of 22.66%, 23.48%, and 24.52% under illuminations of 201.8 lx, 607.8 lx, and 999.6 lx, respectively) bi-facial DSSC for indoor applications [94], using the Y123 dye as a sensitizer. Grätzel and co-workers fabricated a DSSC using XY1b/Y123 as a sensitizer, which exhibited a high PCE (32%) under the illumination of a 1000 lx Osram 930 warm white fluorescent lamp [78]. They created a direct contact between the counter electrode (PEDOT) and dye-impregnated TiO_2_ film of the device. This reduced the diffusion path and significantly enhanced the PCE of the device. Freitag et al. utilized a low-cost hybrid dye sensitizer (XY1 + 5T) in a DSSC [95]. The device exhibited a high PCE (29.2%) under the illumination of a 1000 lx fluorescent lamp.

Some significant results on different DSSCs for indoor applications are summarized in Table 2. From this detailed review, it is evident that DSSCs are attractive options for harvesting indoor light energy. A PCE ~32% under the illumination of a 1000 lx fluorescent light has been achieved in a laboratory environment from an XY1b/Y123 co-sensitizer-based DSSC. For the last few years, researchers have been attempting to develop new Ru-free dyes to avoid inconvenience due to the lack of availability of Ru in the near future. Some researchers have also attempted to develop new dyes with a higher optical energy bandgap (appropriate for indoor light energy harvesting) through the molecular engineering of dye molecules. Furthermore, the development of alternative low-cost and less-reactive materials for the counter electrode has been attempted. The combination of the efforts of various research groups and the PV cell industry has already made indoor DSSC PV modules available in the market. However, these efforts are not sufficient. Further research is necessary for the development of low-cost, environmentally stable new dyes with a high extinction coefficient. Additionally, a special focus on the development of suitable alternatives to Pt counter electrodes to minimize the dark current and charge carrier recombination is highly recommended.

### 3.3. Solar Cells Based on Organic Materials for Indoor Applications

**Figure 7:**
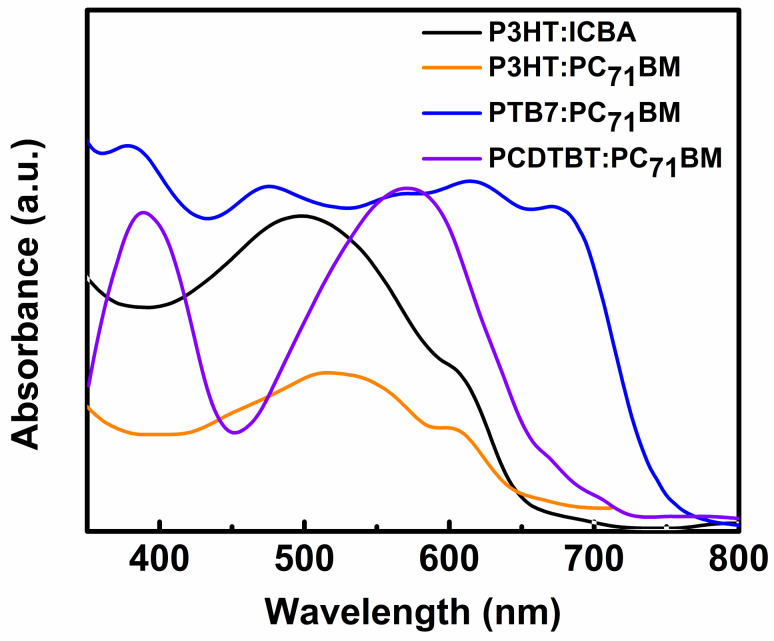
Absorption spectra of some widely used active materials in OSCs for indoor applications. (Reproduced from [96,97,98,99], with permission from AIP Publishing, 2013, AIP Publishing, 2013, and RSC Publishing, 2011 respectively).

Similar to DSSCs, solar cells based on organic materials are promising for indoor applications. Several years after the first development of OSCs, we have achieved an efficiency of approximately 17.4% for outdoor applications (NREL best research cell efficiency table). However, when these devices operate under low-intensity artificial light, their efficiency dramatically increases [43] due to good spectral matching between their absorption spectrum (Figure 7) and the irradiance spectrum of light sources.

**Figure 8:**
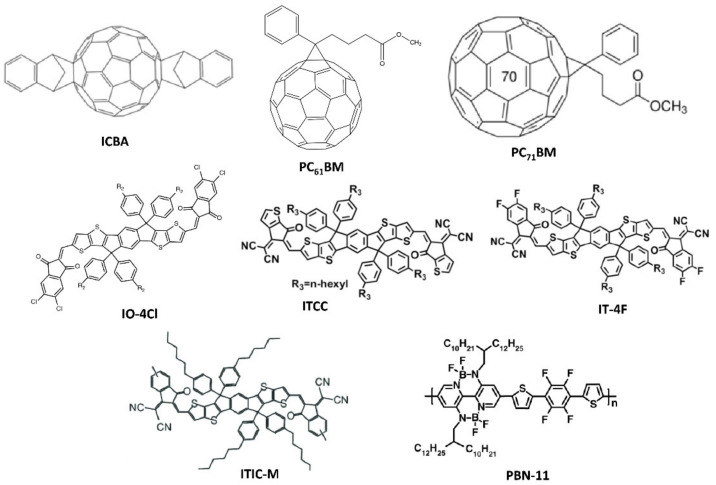
Chemical structures of various widely used fullerene- and non-fullerene-based organic semiconducting acceptor materials in OSCs.

**Figure 9:**
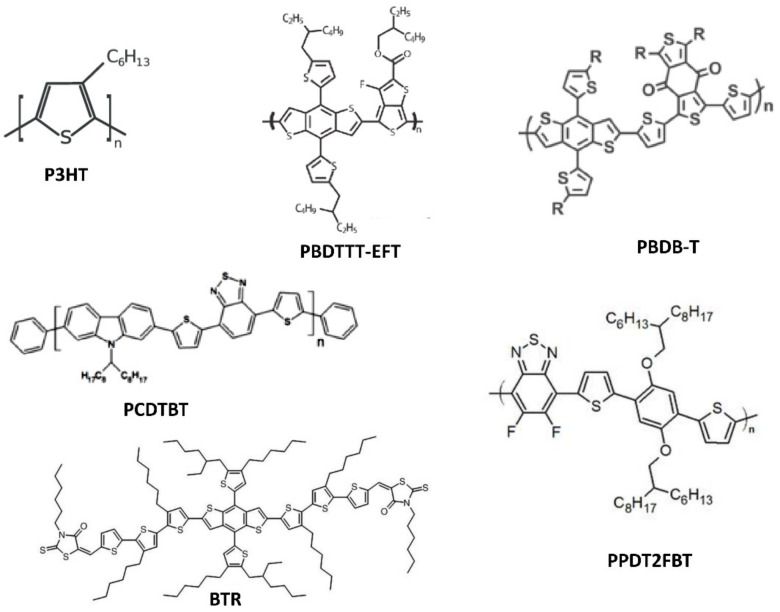
Chemical structures of various widely used organic semiconducting donor materials for OSCs.

Owing to their low cost, good mechanical flexibility, low thickness, and favorable optical energy band gap, OSCs are one of the most promising PV devices for indoor light energy harvesting [100]. OSCs have six main components, namely, substrate, anode, hole transport layer (HTL), active layer, electron transport layer (ETL), and cathode. Each layer of the device plays a crucial role in its operation. In 2010, Minnaert and Veelaert first reported the applicability of OSCs for indoor applications [101]. After 10 years, a PCE more than 26% has been achieved for an OSC operating in low-intensity indoor light [102]. In the last decade, various research groups have made significant efforts toward the development of efficient, stable, long-lasting, and low-cost OSCs for indoor applications [43]. The active layer is one of the most important components of the OSC. It is formed through the mixing of p-type donor and n-type acceptor semiconducting materials. This mixing forms a bulk heterojunction (BHJ). The size distribution of the semiconductors should be within a nanometer range because the exciton diffusion length of the organic semiconducting material is within this range. Various organic semiconducting materials have been used as donor and acceptor materials for indoor OSCs so far. Owing to their satisfactory charge transport properties and desirable absorption spectra, various fullerene-based organic semiconductors, such as indene-C60 bisadduct (ICBA), phenyl-C61-butyric-acid-methyl ester (PC_61_BM), and [6,6]-phenyl C71-butyric acid methyl ester (PC_71_BM), have been initially used as acceptor materials for indoor OSCs (Figure 8) [43]. Subsequently, some non-fullerene organic semiconducting materials, such as IO-4Cl, IT-4F, ITCC, ITIC-M, TPDI2N-EH, PBN-11, and ITIC-F, have been introduced as acceptors, owing to their better response to low-intensity indoor light (Figure 8) [43]. Subsequently, various organic semiconducting polymers and small molecules, such as P3HT, PBDTTT-EFT, PBDB-T, PCDTBT, PPDT2FBT, BTR, DTCPB, and P1 (Figure 9), have been utilized as donor materials of OSCs for indoor applications [43]. 

Accordingly, researchers have employed different techniques for device fabrication. Various aspects, such as the weight ratio of donor and acceptor materials, the thickness and surface morphology of the film, and the nature of the solvent, should be monitored for the formation of the active layer. This is because every aforementioned aspect has a strong impact on the overall performance of OSCs for indoor applications. In addition to the active layer, the HTL and ETL play a crucial role in an OSC. These two layers act as bridges between the active layer and the electrode of the device. They help reduce the charge carrier recombination within the device. Therefore, in the past few years, considerable efforts have also been devoted toward the development of low-cost, less reactive, processable, and environmentally stable semiconducting materials to be used as the HTLs and ETLs of OSCs [12]. During the early development of indoor OSCs, Steim et al. determined that the performance of an OSC is strongly dependent on its shunt resistance when the device is operating under the illumination of low-intensity indoor light [103]. Mori et al. fabricated an OSC based on a PTB7-Th donor and PC_70_BM acceptor and determined that the PCE of the device was approximately 10.55% under the illumination of a 186 lx LED lamp [39]. They proposed that the device could exhibit a PCE approximately 21% under realistic conditions. In 2016, Lee et al. tested OSCs based on three different semiconducting donors, i.e., P3HT, PCDTBT, and PTB7, and a fullerene-based acceptor PC_71_BM under the illumination of low-intensity indoor light [104]. They observed that all the devices showed better performance under low-intensity light than under sunlight and, among these three devices, the OSC based on a PCDTBT:PC_71_BM active material exhibited the highest PCE of 16.6%. As the thickness of the active layer of an OSC plays a crucial role in the overall performance of the device as discussed earlier, various researchers have attempted to optimize the thickness of the active layer of different OSCs to achieve a higher PCE [105]. For example, Kim et al. showed that a P3HT:ICBA-based OSC with an optimized thickness of the active layer could exhibit better performance under the illumination of an LED light [40,106]. They performed an optical simulation study using the finite-difference time-domain (FDTD) method to reduce the experimental cost and wastage of time. Recently, Shim and co-workers improved the PCE of a P3HT:ICBA-based inverted organic PV (OPV) under the illumination of an LED lamp by modifying the indium tin oxide (ITO) surface with ethoxylated polyethylenimine (PEI) [107]. They observed that the device showed a high shunt resistance at the optimized thickness of the PEI layer, which reduced the leakage current in the device and resulted in a high PCE of 13.9%. 

Moreover, the same research group developed a highly efficient (PCE = 12.3%) and mechanically stable flexible OSC for indoor applications, through the utilization of a quasi-amorphous ZnO/Ag/ZnO transparent electrode using P3HT:ICBA as the active material [108]. In addition to the use of binary BHJ active materials, some researchers attempted to use multi-donor and -acceptor BHJs to achieve a better PCE through the improvement of the absorption window [13,22,109,110]. For example, Yin et al. could achieve PCE over 20% using a ternary OSC based on a PCDTBT:PDTSTPD:PC_71_BM active material [22]. Following this protocol, Kim et al. later developed a quaternary BHJ using two donors (PCDTBT and PTB7) and two acceptors (PC_61_BM and PC_71_BM) [13]. The quaternary indoor OPV device exhibited a PCE greater than 10% under the illumination of a 500 lx LED lamp. Vincent et al. demonstrated the exact role of quaternary mixtures during the operation of an OSC for indoor applications by conducting an FDTD-based optical simulation study [111]. They observed that there was strong absorption of the quaternary OSC at an optimized thickness of its active layer under the illumination of a low-intensity indoor light, owing to the high oscillations at an ideal short-circuit current density (J_sc,ideal_). In addition, Cui and co-workers achieved PCE above 26% under an LED lamp (1000 lx) through the utilization of a wide-bandgap non-fullerene acceptor IO-4Cl and a polymer donor PBDB-TF [102]. They also demonstrated an accurate way of calculating the PCE of an indoor PV cell. The irradiance power intensity of a non-standard illuminating agent should be measured cautiously. The incident light power is generally measured with a lux meter, which is not suitable for determining the power intensity of indoor light. Commercially available lux meters are equipped with spherical probes, which are significantly different from planar PV cells; thus, they are not suitable for evaluating the power intensity of incident light. Therefore, Cui and co-workers suggested that the power intensity of light should be measured with a reliable spectrometer and that the integral current density calculated from the external quantum efficiency curve and the incident light spectrum should be compared with the current density value obtained from the current density–voltage (J–V) measurements [102]. Some significant results on different OSCs for indoor applications are summarized in Table 3. From this review study, it is evident that several studies have been conducted during the past decade for the development of indoor OSCs. Different types of device fabrication technologies, donor-acceptor materials, HTLs, ETLs, and substrates have been employed to achieve better performance. For example, a transition from fullerene to non-fullerene acceptors for ISCs has been observed in the past few years as the latter type of acceptors have higher photon absorption ability in the visible range and tunable molecular and optical bandgap energies. A PCE ~27% under the illumination of a 1000 lx LED light has been achieved in a laboratory environment from an OSC based on an organic polymer donor (PBDB-TF) and non-fullerene (IO-4Cl) acceptor. Different techniques for increasing the optical band gap of active materials have been developed so far. Different device parameters have also been optimized through the combination of experimental and simulation studies. However, this is not sufficient and further investigation is required in this regard. In particular, the lifetime of the OPV is significantly lower than that of its inorganic counterpart. ISCs must show a minimum of 80% of their initial PCE for more than 10 years to compete with the general batteries used in IoT devices. On the other hand, their production cost is high. Therefore, more studies are necessary to improve the lifetime and reduce the production cost of OSCs to enhance their acceptability in the commercial sector.

### 3.4. Solar Cells for Indoor Applications Based on Perovskite Materials

**Figure 10:**
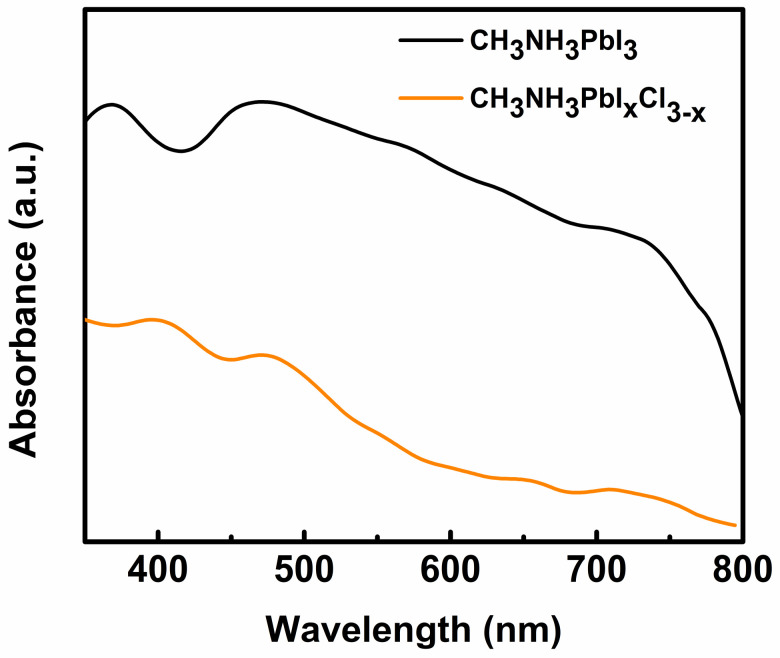
Absorption spectra of some widely used active materials in PVSCs for indoor applications. (Reproduced from [115], with permission from Elsevier, 2018.

During the past eight years, organic/inorganic hybrid perovskite materials have attracted considerable interest owing to their synergic physicochemical properties. Their low bandgap (~1.6 eV) and good optical and semiconducting properties motivated researchers to develop fourth-generation solar cells. Within a few years after their first development, PVSCs achieved a PCE of 25.2% in a laboratory environment under the 1-sun condition (NREL best research cell efficiency table). On the other hand, their long charge carrier diffusion length, panchromatic light absorption ability (Figure 10) within the visible spectrum range, high carrier mobility, and low exciton binding energy have rendered them potential candidates for low-intensity light energy harvesters [112]. However, few studies have investigated the performance of PVSCs in an indoor environment thus far. In 2015, Chen et al. tested a PVSC based on a CH_3_NH_3_PbI_3−x_Cl_x_ active material under the illumination of low-intensity indoor light for the first time and achieved PCE approximately 27% at optimized conditions [113]. The perovskite material in a PVSC is generally sandwiched between the HTL and the ETL. Therefore, the overall performance of a PVSC is strongly dependent on the physicochemical properties of the materials used to form the three layers [114]. 

Raifuku and co-workers studied the characteristics of PVSCs under the illumination of low-intensity indoor light [116]. Moreover, they investigated the difference between the internal states of the solar cells for both outdoor and indoor lights via impedance spectroscopy. They observed that a planar-type solar cell could provide better performance than a meso-structured solar cell for indoor applications, owing to its high shunt resistance. In 2017, Lucarelli and co-workers first reported the fabrication of a flexible PVSC for indoor applications [17]. They fabricated the device on an ITO-coated flexible polyethylene terephthalate substrate.Their flexible PVSC based on a CH_3_NH_3_PbI_3–x_Cl_x_ perovskite semiconductor and a spiro-MeOTAD HTL exhibited PCEs 10.8% and 12.1% under the illumination of 200 lx and 400 lx LED lamps, respectively. Although their flexible PVSCs exhibited lower efficiency than a rigid device based on the same material, their flexible characteristic, low weight, and ultra-thin nature render them more attractive for indoor applications. Dagar et al. could increase the PCE of a CH_3_NH_3_PbI_3_–based planar indoor PVSC by using SnO_2_/MgO composites as the ETLs [117]. They achieved a PCE of 26.9% under the illumination of a 400 lx LED lamp. In addition to the development of different semiconducting materials for ETLs, some researchers have attempted to improve the performance of PVSCs for indoor applications through the development of different p-type semiconducting materials as HTLs. For example, Jagadamma et al. developed an efficient p-i-n hybrid indoor PVSC by using NiO as the HTL [118]. Their device exhibited a PCE of 23% under the illumination of a fluorescent lamp having an irradiance power intensity of 0.32 mW/cm^2^. Moreover, Mathews and co-workers successfully used a wide-bandgap indoor PVSC as a power source for a backscatter sensor [119]. They connected three PVSCs in a series configuration to create a module that harvested power of 14.5 μW from a compact fluorescent illumination of 0.16 mW/cm^2^ with a PCE of 13.2%. Some significant results on different PVSCs for indoor applications are summarized in Table 4. From the review study, it is evident that PVSCs have become a highly promising type of PV cells for indoor light harvesting within a few years due to their remarkable optoelectronic properties. A PVSC based on a CH_3_NH_3_PbI_3_ active material has already achieved PCE ~35% under the illumination of a 1000 lx fluorescent light in a laboratory environment [120]. This is a promising result for the commercialization of indoor PVSCs in the near future. However, more investigation on aspects, such as lifetime, toxicity, environmental stability, and device fabrication process is necessary. In this regard, development of new HTLs, ETLs, substrates, and perovskite materials with synergic physicochemical properties along with the optimization of different device parameters can be helpful toward the commercialization of new-generation indoor PVSCs. 

## 4. Future Prospects 

**Figure 11:**
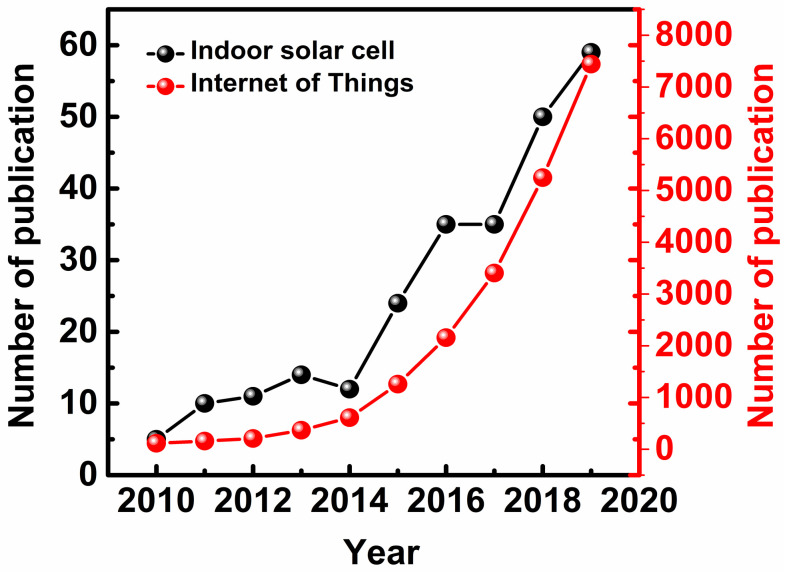
Number of publications on the topics named indoor solar cell and IoT per year (2010–2020) (Source: Web of Science).

With the rapid growth of the IoT, the demand for indoor solar cells has steadily increased (Figure 11). Some companies have already introduced indoor solar cells to the market.

Panasonic, Powerfilm, and Solemshave have commercialized indoor solar cell modules based on a-Si. GCell has already launched indoor modules in the market and Alta Devices and Lightricity are selling indoor solar cells based on III–IV semiconducting materials [1]. Although PVSCs and OSCs have already demonstrated considerable potential for indoor applications, they have not been commercialized thus far. More improvements in terms of various aspects are necessary. First, the lifetimes of OSCs and PVSCs are considerably shorter than those of commercially available ISCs and DSSCs. It is not possible to change the PV modules frequently for powering an IoT network. Therefore, improvement of the lifetime of PV devices through the development of new, environmentally stable constituent materials in the near future is highly recommended. From the extensive research study, it is also observed that there is no universally accepted measurement protocol for ISCs. Hence, it is difficult to accurately estimate the efficiency of a device under the illumination of different artificial light sources. This situation is not suitable for the commercialization of new indoor PV devices. Therefore, the development of a universally accepted measurement protocol similar to the 1-sun condition is urgently required. The theoretically calculated maximum PCE of an ideal ISC is ~50%. However, to date, we have only achieved a PCE of 30% in a laboratory environment; thus, there is a huge gap between the real and expected values. Hence, the development of new active materials having an optimized energy bandgap (~1.9 eV), high diffusion length, low charge recombination probability, and the synergic surface property is highly recommended. Moreover, the development of different device architectures, thicknesses of different layers, and more efficient device fabrication technologies for different ISCs is required. The production cost of commercially available indoor PV modules is another key factor; therefore, the development of new cost-effective constituent materials for ISCs and a convenient, time-saving, efficient, and cost-effective device fabrication technology in the near future is also necessary. A highly efficient indoor PV module with good mechanical flexibility must have a high demand; however, few researchers have attempted to develop such a PV device for indoor applications so far. Moreover, the PCE values of ISCs are not as good as those of their rigid counterparts and their mechanical stability needs to be improved for commercialization. Therefore, intensive studies in this field are required. Most ISC modules will be used for powering the different component devices of IoT networks, which are mostly wearable. Thus, the biocompatibility of these ISC modules should be ensured and development of eco-friendly and biocompatible ISC components in the near future are highly recommended. We believe that ISCs will reach commercial-scale production within a few years if they satisfy the aforementioned requirements.

## 5. Conclusions

In this study, we performed a detailed review of the development of various solar cells for indoor applications. It is thus observed that although ISCs are dominating the outdoor solar cell market, they are not suitable for use as indoor light-harvesting units because of their low bandgap energy and poor mechanical flexibility. In contrast, the active materials of DSSCs, OSCs, and PVSCs show unique properties, such as high-energy bandgap, good spectral matching with the irradiance spectrum of low-intensity indoor light, tunable bandgap energy, mechanical stability, solution processability, and thin-film formability, which render them promising candidates for use as indoor light energy harvesters. In the last decade, numerous DSSCs, OSCs, and PVSCs have been fabricated and tested for indoor applications. Researchers have already achieved a satisfactory PCE of these solar cells in the indoor environment. In fact, DSSCs have already been commercialized. However, these results are still insufficient, and intensive studies are required to improve their commercial acceptability. For instance, the lifetime of OSCs and PVSCs is considerably shorter than that of ISCs. An ISC that retains more than 80% of its initial PCE value even after 10 years of its fabrication needs to be developed to compete with the currently used power sources (general battery) in IoT devices. In addition, the biocompatibility of these devices should be improved through the development of new, non-toxic semiconducting materials. Moreover, the simple processability of different constituent materials of an ISC module is highly desirable for their commercialization. Hence, the development of new, highly processable PV materials is recommended. Also, the different device parameters such as short circuit current density, open circuit voltage, and fill factor should be optimized to achieve significantly better performance. We believe that this systematic review will be helpful to researchers attempting to overcome the various challenges toward the commercialization ISCs in the near future.

## Figures and Tables

**Table 1:** Summary of previously reported studies on different ISCs for indoor applications.

Reference Number	Active Material	Light Source	Luminance (Lux)	MPD ^2^ (µW/cm^2^)	PCE ^3^ (%)
[14]	a-Si	FL ^1^	200	8.10	-
[14]	a-Si	FL	1000	46.50	-
[14]	a-Si	LED	200	9.40	-
[14]	a-Si	LED	1000	46.40	-
[14]	GaAs	FL	200	13.80	-
[14]	GaAs	FL	1000	80.50	-
[14]	GaAs	LED	200	16.60	-
[14]	GaAs	LED	1000	92.20	-
[14]	GaInP	FL	200	15.60	-
[14]	GaInP	FL	1000	92.60	-
[14]	GaInP	LED	200	17.60	-
[14]	GaInP	LED	1000	87.20	-
[15]	Al_0.2_Ga_0.8_As	LED	580	>100	21.10
[53]	a-Si	FL	100	-	9.10
[54]	a-Si	-	-	-	6.00–8.00
[55]	a-Si	LED	500	-	9.60
[56]	CdS/CdTe	Halogen	-	-	8.00
[57]	CIGS	-	-	-	3.00
[58]	CIGS	FL	100	2.10	5.80
[58]	CIGS	FL	500	14.90	8.30
[58]	CIGS	FL	1000	33.70	9.40
[58]	CIGS	Halogen	100	2.90	7.30
[58]	CIGS	Halogen	500	20.00	10.20
[58]	CIGS	Halogen	1000	44.90	11.50
[62]	CIGS	LED	-	-	2.64

^1^ FL: Fluorescent lamp; ^2^ MPD: Maximum power density; ^3^ PCE: Power conversion efficiency.

**Table 2:** Summary of previously reported studies on DSSCs for indoor applications.

Reference Number	Sensitizer	Light Source	Luminance (Lux)	MPD ^2^ (µW/cm^2^)	PCE (%) ^3^
[71]	N719	Philips TLD 840 FL	250	69.80	-
[72]	N719	FL ^1^	1533	13.91	6.05
[73]	N719	T5 FL	200	7.29	11.38
[73]	Z907	T5 FL	200	7.84	12.23
[74]	SK6	T5 FL	6000	366.57	19.46
[74]	CW10	T5 FL	6000	395.46	20.95
[74]	SK6 + CW10	T5 FL	6000	426.10	22.58
[74]	N719	T5 FL	6000	435.86	23.43
[75]	Y1A1	LED	350	-	19.50
[76]	TF-tBu-C3F7	T5 FL	2400	-	20.37
[76]	TF-tBu-C3F7	LED	2400	-	16.05
[78]	XY1b + Y123	930 Osram FL	500	132.00	30.80
[78]	XY1b + Y123	930 Osram FL	1000	283.00	31.80
[79]	L350	FL	1000	-	28.40
[80]	MD4	T5 FL	6000	-	8.62
[80]	MD5	T5 FL	6000	-	23.17
[80]	MD6	T5 FL	6000	-	16.86
[80]	MD7	T5 FL	6000	-	27.17
[80]	N719	T5 FL	6000	-	27.64
[85]	N719	FL	200	-	-
[92]	SK7	T5 FL	6000	335.00	17.70
[92]	YD2	T5 FL	6000	340.00	20.00
[92]	SK7	LED	6000	277.00	15.40
[92]	YD2	LED	6000	296.00	16.50

^1^ FL: Fluorescent lamp; ^2^ MPD: Maximum power density; ^3^ PCE: Power conversion efficiency.

**Table 3:** Summary of previously reported studies on different OSCs for indoor applications.

Reference Number	Active Material	Light Source	Luminance (Lux)	MPD ^2^ (µW/cm^2^)	PCE ^3^ (%)
[13]	PCDTBT:PTB7:PC_61_BM:PC_71_BM	LED	500	18.00	10.60
[22]	PCDTBT:PC_71_BM	FL ^1^	300	12.20	16.50
[22]	PCDTBT:PDTSTPD:PC_71_BM	FL	300	15.40	20.80
[23]	P1:PC_71_BM	LED	300	14.86	19.15
[23]	PCDTBT:PC_71_BM	LED	300	14.53	18.72
[39]	PTB7-Th:PC_70_BM	LED	890	42.6	11.63
[39]	PTB7-Th:PC_70_BM	LED	1861	76.2	10.55
[102]	PBDB-TF: IO-4Cl	LED	1000	78.80	26.00
[104]	P3HT:PC_71_BM	FL	300	4.80	5.80
[104]	PCDTBT:PC_71_BM	FL	300	13.90	16.60
[104]	PTB7:PC_71_BM	FL	300	12.20	14.60
[105]	PPDT2FBT:PC_70_BM	LED	1000	44.80	16.00
[106]	P3HT:ICBA	LED	1000	-	5.40
[107]	P3HT:ICBA	LED	500	22.78	13.40
[108]	P3HT:ICBA	LED	500	20.57	12.10
[109]	PTB7-Th:(PBDB-T:PC70BM:ITIC-Th	LED	1000	-	15.46
[109]	PTB7-Th:(PBDB-T:PC70BM:ITIC-Th	FL	1000	-	14.69
[110]	PTB7:PC_71_BM:EP-PDI	LED	500	-	15.68

^1^ FL: Fluorescent lamp; ^2^ MPD: Maximum power density; ^3^ PCE: Power conversion efficiency.

**Table 4:** Summary of previously reported studies on different PVSCs for indoor applications.

Reference Number	Active Material	Light Source	Luminance (Lux)	MPD ^2^ (µW/cm^2^)	PCE (%) ^3^
[17]	CH_3_NH_3_PbI_3–x_Cl_x_	LED	200	-	10.80
[17]	CH_3_NH_3_PbI_3–x_Cl_x_	LED	400	-	12.10
[112]	CH_3_NH_3_PbI_3_	LED	200	12.36	-
[112]	CH_3_NH_3_PbI_3_	LED	400	28.03	-
[112]	CH_3_NH_3_PbI_3_	LED	800	63.79	-
[112]	CH_3_NH_3_PbI_3_	LED	1600	147.74	-
[112]	CH_3_NH_3_PbI_3_	Halogen	200	56.43	-
[112]	CH_3_NH_3_PbI_3_	Halogen	400	100.97	-
[112]	CH_3_NH_3_PbI_3_	Halogen	800	187.67	-
[112]	CH_3_NH_3_PbI_3_	Halogen	1600	376.85	-
[113]	CH_3_NH_3_PbI_3−x_Cl_x_	FL ^1^	100	-	20.90
[113]	CH_3_NH_3_PbI_3−x_Cl_x_	FL	600	-	25.10
[113]	CH_3_NH_3_PbI_3−x_Cl_x_	FL	1000	-	26.30
[117]	CH_3_NH_3_PbI_3_	LED	400	-	26.90
[118]	CH_3_NH_3_PbI_2.9_Cl_0.1_	FL	-	73.60	23.00
[118]	CH_3_NH_3_PbI_2.9_Cl_0.1_	LED	-	457.60	20.80

^1^ FL: Fluorescent lamp; ^2^ MPD: Maximum power density; ^3^ PCE: Power conversion efficiency.
